# Letter to Editor: ‘Identification of patients with favorable prognosis after resection in intermediate-stage hepatocellular carcinoma’

**DOI:** 10.1097/JS9.0000000000001681

**Published:** 2024-05-20

**Authors:** You-Jian Xu, Hui-Ming Pang

**Affiliations:** Department of General Surgery, Tiantai People’s Hospital of Zhejiang Province (Tiantai Branch of Zhejiang Provincial People’s Hospital), Hangzhou Medical College, Taizhou, Zhejiang, People’s Republic of China

*Dear Editor,*

We read the recent study by Lee *et al*.^1^ with great interest. The study, based on a multicenter database, provided a comprehensive analysis of intermediate-stage hepatocellular carcinoma (HCC) in a large cohort of 1686 patients who underwent liver resection or trans-arterial chemoembolization (TACE). This study demonstrated that alpha-fetoprotein ≥5.0 ng/ml, ALBI grade ≥2, tumor number ≥3, and maximal tumor size ≥5 cm were significant risk factors for 2-year recurrence-free survival (RFS) in the resection group. Meanwhile, a newly developed prediction score, the surgery risk score in BCLC-B (SR-B score), had very good predictive accuracy for 2-year RFS after the resection in intermediate-stage HCC. While this study offers valuable clinical insights for selecting treatment strategies for intermediate-stage HCC, there are still several aspects that warrant further discussion.

Firstly, patients diagnosed with intermediate-stage HCC who underwent surgery (with histologically confirmed complete removal of tumor tissue) or TACE [with achieved complete response (CR)] as their first line of treatment were recruited in the present study. The primary endpoint of this study, as noted by the authors, was RFS, which is commonly utilized in curative treatments, particularly hepatectomy and liver transplantation. The imaging findings indicating CR after TACE do not necessarily imply that the postoperative pathology has achieved CR^2^, resulting in the 2-year RFS of the TACE group being poorer in comparison to the surgical group. Consequently, employing RFS as a study endpoint for those who did not truly achieve CR may reduce the reliability of the study.

Secondly, there is no doubt that surgery is superior to TACE in selected patients with intermediate-stage HCC. As a retrospective study, it is inevitable that there will be selection bias in the treatment of patients. To minimize baseline disparities, the authors employed propensity score matching (PSM). From the baseline table, we can see that the tumor size is 5 cm, and the number is 3, which is the cut-off value. However, some studies have shown that random binary cutoff for both tumor size and number can reduce statistical power, which may not truly reflect the tumor burden between the two groups^3^. Therefore, despite the use of PSM, it is difficult to balance the two groups of patients. The tumor burden score (TBS) is derived from precise calculations, considering tumor size and number as continuous variables. TBS has the potential to serve as a reliable tool for stratifying the prognosis of patients with colorectal liver metastases or HCC undergoing resection, which may be more appropriate for this study^4^.

Thirdly, although the authors did mention the proportion of hepatitis patients in both groups, they did not furnish details on antiviral treatment administered to these patients. Antiviral therapy plays an important role in delaying disease progression and mitigating HBV-related HCC recurrence. Consequently, uncertainty remains regarding the potential impact of antiviral therapy disparities between the two groups on long-term outcomes^5^.

In summary, clarification regarding the above-mentioned omissions would greatly solidify the conclusions of the study by Lee and colleagues.

## Ethical approval

Not applicable.

## Consent

Not applicable.

## Sources of funding

This research did not receive any specific grant from funding agencies in the public, commercial, or not-for-profit sectors.

## Author contribution

Y.-J.X.: drafted the article; H.-M.P.: revised the manuscript and approved the final version.

## Conflicts of interest disclosure

The author declares no conflict of interest.

## Research registration unique identifying number (UIN)

1. Name of the registry: not applicable.
2. Unique identifying number or registration ID: not applicable.
3. Hyperlink to your specific registration (must be publicly accessible and will be checked): not applicable.

## Guarantor

Hui-Ming Pang, Tiantai People’s Hospital of Zhejiang Province (Tiantai Branch of Zhejiang Provincial People’s Hospital), Hangzhou Medical College, Taizhou, Zhejiang, China (E-mail: panghuiming72@hotmail.com)

## Data availability statement

All data generated or analyzed during this study are included in this article. The data are available from the corresponding author upon reasonable request.

## Provenance and peer review

Not applicable.
